# Vaults, heroes, and enemies: a multimodal approach to poster propaganda in the *Fallout* series

**DOI:** 10.3389/fpsyg.2025.1576282

**Published:** 2025-06-06

**Authors:** Dušan Aleksić, Miloš Tasić, Dušan Stamenković

**Affiliations:** ^1^Faculty of Philosophy, University of Niš, Niš, Serbia; ^2^Faculty of Mechanical Engineering, University of Niš, Niš, Serbia; ^3^School of Culture and Education, Södertörn University Stockholm, Huddinge, Sweden

**Keywords:** propaganda, posters, multimodality, video games, *Fallout*

## Abstract

The *Fallout* game series has utilized in-game posters as both decorative and narrative elements, which have helped it shape its post-apocalyptic world. These posters often serve as (satirical) propaganda and reflect the societal structures, ideologies, and conflicts of the game’s dystopian setting. This study analyzes all propaganda-themed posters from six *Fallout* role-playing video games (1997–2018) to examine their multimodal features and propaganda techniques. Using a multimodal framework combined with propaganda studies, we explore how text-image relations, figures of speech, typography, color schemes, and iconography interact with propaganda strategies such as *Appeal to emotion* and *fear*, *Name-calling, Glittering generalities*, and *Band wagon*. Our findings reveal that text-dominant posters and sans-serif fonts are the most frequent design choices. Metonymy is the most used figure of speech, and it often reinforces ideological symbols, while red and blue emerge as dominant colors, frequently associated with military or nationalistic themes. The study also identifies strong correlations between specific propaganda techniques and multimodal features, demonstrating how visual and textual elements work together to achieve persuasive impact. The study situates *Fallout’s* posters within a broader context and provides insight into how video games appropriate and adapt real-world propaganda strategies and forms of political messaging.

## 1 Introduction

Since early 2024, the *Fallout* game series (Interplay Entertainment, 1997; Black Isle Studios, 1998; Bethesda Game Studios, 2008; Bethesda Game Studios, 2015, 2018; Obsidian Entertainment, 2010) has experienced a significant resurgence in popularity, largely attributed to the release of an Amazon-produced (Amazon Studios, 2024) television adaptation. This renewed interest has brought in a wave of new players, as well as rekindled the enthusiasm of longtime fans (Maas, 2024). The growing player base, coupled with the expansive multimedia presence of the *Fallout* universe, confirms the enduring influence of this post-apocalyptic franchise. In this situation, we believe it is worth examining the visual elements that have contributed to its wide appeal, particularly those that exist within its rich game-world. One of the features of the *Fallout* series that sets it apart is its extensive use of posters as a world-building tool. These posters serve a dual function: they are both decorative elements and carriers of messages which provide players with crucial insight into the societal structures, ideologies, and conflicts that exist within the game’s post-apocalyptic dystopian setting. They are essentially background pieces of knowledge about the game world. The *Fallout* world is shaped by its history of nuclear conflict, technological innovation, and survivalist philosophies, and the posters within the game reflect these. They portray everything from recruitment efforts for military factions to advertisements for Vault-Tec, the organization responsible for the construction of the network of underground vaults where survivors reside, and serve as a form of in-game propaganda, presenting idealized visions of safety, loyalty, and survival in a post-apocalyptic society. Although *Fallout* posters may not directly affect the player’s decisions within the gameplay – for instance, some players may not encounter or notice many of them, they are a potential instrument of immersion for the reasons outlined above. This paper aims to analyze posters which contain elements of propaganda from the *Fallout* series through combining multimodality with propaganda studies.

## 2 *Fallout* and researching *Fallout*

*Fallout* is a long-standing series of role-playing games (RPGs) set in a post-apocalyptic world shaped by the aftermath of a nuclear war, later revealed to be the Great War, a result of the Sino-American War between China and the USA. First launched in 1997, with the latest game released in 2018, the franchise has since expanded into multiple games and other media. It presents a retro-futuristic vision of the world, blending 1950s aesthetics with speculative science fiction (see Lafleuriel, 2019). The series is known for its dark humor, sarcasm, moral dilemmas, and richly detailed game-world, where players roam a vast wasteland devastated by war (see Chandler, 2015; Schulzke, 2009). Set primarily in the remains of the United States after a global nuclear conflict in the mid-21st century, the *Fallout* series explores a society that has collapsed into chaos, where survivors struggle to rebuild amid radiation, mutated creatures, and opposing factions (see Hodgson and von Esmarch, 2015). Some remnants of pre-war civilization still exist, particularly through the Vaults – underground shelters built by the government to protect a selected few from the nuclear fallout. The posters that we will try to analyze here are part of the above-mentioned *Fallout* universe’s retro-futuristic aesthetic.

The *Fallout* video game series has been assessed from various perspectives in several academic ventures. Iversen (2012) uses *Fallout 3* in proposing a broad conceptualization of “challenge” as a basis for a holistic analysis of digital games. She explores the dual nature of challenge as both demanding (uncertain) and stimulating (indeterminate) and discusses how digital games engage players on both mechanical and aesthetic levels. McClancy (2018) uses Baudrillard’s theories of simulacra and simulation to analyze the *Fallout* game series’ retro-futuristic setting and argues that the games present a nostalgic simulacrum of the 1950s Cold War era, criticizing the blind faith in technology from that time. As posters in the *Fallout* games are part of this experience, we believe that they are included within this as well. Gonzales’s thesis (2010) examines *Fallout 3* with regard to how it mediates cultural perceptions of the past, memory, and history, and challenges assumptions about technology and progress. Domsch (2015) examines the relationship between video games and utopian or dystopian themes and explains how video games, as rule-bound systems, can represent societal structures and player agency. Similarly, Kemmer’s (2014) approach explores the political, ethical, and narrative dimensions of *Fallout 3* and focuses on the game’s interactive nature, which allows players to navigate moral dilemmas in a world without (or with unusual) societal structures. The open-world structure allows players to experiment with their moral decisions, which Kemmer relates to the game’s broader philosophical commentary on moral ambiguity. The topics of morality and decision-making are central to Schulzke’s (2009) and Casas-Roma and Arnedo-Moreno’s (2019) approach to *Fallout*, too.

## 3 Theoretical framework

The theoretical framework is built on a triangulation of two key components: multimodality in video game posters on the one hand and propaganda functions and techniques on the other. The first component draws on multimodality and focuses on how visual, linguistic, and spatial modes interact in *Fallout* posters to create meaning. The second component examines classic and modern propaganda techniques, exploring how these posters function not just as immersive elements but also as persuasive tools that reflect real-world and game-world propaganda strategies. In this section, we are going to introduce both of these components and, in this way, see why we have opted for a mixed method in the present study.

### 3.1 Video games, posters and multimodality

A multimodal approach has been applied to video games on several occasions, given that video games are particularly complex examples of multimodal artifacts because they incorporate diverse communication modes. This complexity is further increased by the ongoing advancement of gaming technologies, which continue to introduce new layers of meaning-making. Within a single game, players engage with various semiotic resources, including animations, images, music, language, interactive tools, and haptic feedback, all of which combine to create immersive and rich experiences. Analyzing video games from a multimodal perspective enables researchers to investigate how these elements combine to generate meaning. However, it also presents significant challenges in identifying and categorizing the numerous elements at play, particularly in games that simulate real-life settings within 3D worlds (Bateman et al., 2017; Wildfeuer and Stamenković, 2020).

Multimodal approaches to video games have, so far, been diverse and focused on areas such as video game discourse (Machin and van Leeuwen, 2007; Ensslin and Balteiro, 2019; Stamenković et al., 2017) and discourse structure (Wildfeuer and Stamenković, 2022; Stamenković and Wildfeuer, 2024), the player experience (Toh, 2018), multimodal semiotics (Hawreliak, 2018), stylistics (Stamenković, 2022), and screen composition (Stamenković and Jaćević, 2019). Although empirical research into the multimodal aspects of video games is still in its early stages, recent studies have started mapping the semiotic interactions within games more empirically (Stamenković and Wildfeuer, 2021). These studies occasionally draw upon research from outside the discipline of multimodality, but they still intersect with it (see, e.g., Aarseth, 2014; Aarseth, 1997; Bogost, 2007; Ensslin, 2012; Juul, 2005; Lemke, 2002).

Our study surveys *Fallout* posters, which in many regards resemble real-life posters (see, e.g., Lewis, 2004; Seidman, 2008; Taylor, 2013). In fact, the early versions of the game feature copies of real-life (mostly WWII) posters. Given this, the multimodal approach will mostly be based on those studies which focused on combining images with text. These were part of multimodality studies from the very beginning (e.g., Kress and van Leeuwen, 1996) and included publications which offered comprehensive views on how the pictorial and the textual material can work together within a canvas to generate meaning (Bateman, 2008, 2014; Bateman et al., 2017; Hiippala, 2015). Fruitful multimodal approaches to artifacts similar to posters could also be seen in the line of research which has addressed multimodal argumentation (see Stöckl and Tseronis, 2024). Within these approaches, we find calls to make evaluation of multimodal arguments such that it takes into consideration the semiotic resources used to communicate them and the context in which they are produced and interpreted (Tseronis et al., 2024), as well as to define a rhetorical figure as being constructed in the process of linking or interaction of text and image elements (Tseronis and Forceville, 2017; Stöckl and Pflaeging, 2022). Multimodal propaganda posters can rely on several key elements to create meaning, such as color, composition/layout, iconography, typography, slogans, figures of speech (e.g., metaphor and hyperbole), spatial arrangement, text-image relationships, and salience of individual elements/hierarchy of different modes. Given this, we are going to frame our method and analysis to classify the posters using several relevant dimensions based on these elements.

### 3.2 Propaganda functions and techniques

This section introduces propaganda functions and techniques and focuses on posters, given the study’s overall goal. It also links the notions of propaganda and metaphor. War propaganda constitutes a crucial component of propaganda communication. One notable observation is that a central function of propaganda is the mobilization of individuals – not merely as supporters of war, but also as active participants in the recommended ways of life dictated by the prevailing circumstances, which is usually done by addressing their values and ideals (Paddock, 2014, p. 9). Effective mobilization ensures that the public not only accepts conflict as a legitimate solution but also embraces roles assigned to them as contributors to the war effort. For example, propaganda posters from the period of World War I frequently depicted women and children as motivators for men to enlist, whereas non-uniformed men were often viewed as a subject of public derision (Paddock, 2014, p. 10).

While propaganda is often defined as a deliberate effort to influence people’s thoughts and actions (Taylor, 2003, p. 6), its role in wartime extends beyond mere manipulation to encompass a process of negotiation. Those targeted by propaganda must negotiate with power holders to determine the extent of their contributions to the shared objective since, as Taylor (2003, p. 13) suggests, the pressure of society at war makes it easier for one to participate in it than distance oneself from it (seen, for example, in the WWI campaign poster ‘What did you do in the Great War, daddy?’), making sacrifice a central theme of this process. Since propaganda is directed toward emotions and not the rational aspect, another important factor in war propaganda is the use of metaphors that rely heavily on symbolism through which a desired meaning is made. According to Steuter and Wills (2009, p. 3), metaphorical terms are chosen as a specific lexicon of language so as to define words in particular ways and shape the ‘what’ and the ‘how’ of our communication. Combining metaphors with cultural codes can result in a creation of an endless set of emotionally provocative meanings.

Posters, as proven throughout the history, are especially useful for conveying this type of propaganda message because they can nationalize, mobilize, and modernize civilian populations, enabling citizens to see themselves as members of the home front (James, 2009, p. 2). However, posters, seen as powerful devices of visual rhetoric during the times of conflict, do not need to serve as a direct call to action. Commercial posters can also have an implicit mobilization function as advertisers do not only market products but a certain way of life, turning such posters into motivators to fight for what is perceived as normal. Considering that propaganda is a planned activity with clear goals and effects it wants to achieve, proper techniques and sub-techniques need to be implemented, depending on what those goals and effects are. In that sense mobilization can be seen as a propaganda function, metaphors as its tool, while the selection of techniques depends on the specific campaign (meant here in the broader sense, including both short-term and long-term ones). Our classification later is going to be based on the employed propaganda techniques, which is why it is important to introduce them at this point. In 1937, the Institute of Propaganda Analysis defined seven devices of propaganda (Scriver, 2015), which represent the basis for detection, research, and further development of propaganda techniques. These devices are:

*Name-calling* – Giving enemies bad names and using pejorative labels based on their group, nation, race, policies, practices, beliefs, and ideals that do not align with those presented as the only ones acceptable.

*Glittering generalities* – Exploiting “virtue words” such as “truth, freedom, honor, liberty, social justice, public service, the right to work, loyalty, progress, democracy, Constitution defender,” so as to connect the propagandists’ viewpoint with those values.

*Transfer* – Acquiring and privatizing widely accepted symbols (i.e., religious and national ones) for a political purpose.

*Testimonial* – Conclusions are not reached based on arguments but on the credibility of sources and people making statements (e.g., when successful actors and athletes promote a certain product, movement, idea, etc.).

*Plain folk* – Refers to situations when representatives of elites (i.e., president candidates) are placed in a context to which they do not belong for a purpose of public image creation. It is often used in political campaigns when candidates visit rural areas, meet and spend time with ordinary people, do their jobs, etc.

*Card stacking* – A propaganda technique that belongs to the domain of logical manipulation. For the purposes of this technique, information or fragments of information that are true are selected, after which a message with a propaganda purpose is constructed (i.e., Christians believe in God – Muslims believe in God – Christians are Muslims). This is one of the most convenient techniques for creating fake news.

*Band wagon* – The propagandist presents information so that it appears to be already accepted and thus to have people “follow the crowd,” because “everybody’s doing it” (Aleksić and Stamenković, 2021; Scriver, 2015; Sproule, 2001).

In war or war-like environments, propaganda techniques are further refined. One key technique is dehumanization, closely tied to name-calling and band-wagoning. This method portrays enemies as inhuman, undermining moral constraints and justifying violence. As Steuter and Wills (2009, pp. 37–38) observe, by dehumanizing the enemy through verbal and visual metaphoric systems (e.g., linking the enemy to objects or animals, dirt or germs, etc.), acts of violence that would otherwise be forbidden are now allowed and celebrated.

Two additional techniques prevalent in war propaganda fall under appeals: *Appeal to fear* and *Appeal to emotion*. Fear, a basic and manipulable emotion, is most effective when controlled, combining promise and threat. It offers solutions while warning of catastrophic consequences for non-compliance, a principle often used by authoritarian regimes and cults. Shabo (2008, p. 75) notes that fearful people are much more supportive of whatever is needed to protect their lives, be it military spending, secret government programs, or hard-line diplomatic tactics. Appeals to emotion exploit the human tendency to react emotionally before reasoning. This technique aims to provoke and manipulate emotions and avoid intellectual abstractions (Jowett and O’Donnell, 2006, p. 230). Emotions are intensified and shaped, whether positive or negative, where love, for example, can be subdivided into compassion, fondness, obsessive attachment, while fear can be broken down into terror, apprehension, uneasiness (Conserva, 2003, p. 21). In a context of widely used propaganda techniques, red herring (diverting attention, spin) and factoids (spreading rumors with the aim of discrediting a certain person or group) should also be mentioned (Aleksić and Stamenković, 2021, p. 37). Today we can distinguish between more than 100 propaganda techniques. Yet, as far as war propaganda is concerned, its techniques can be easily subsumed under the categories discussed in this chapter, which will be used in our classification, and therefore will be part of our methodology.

### 3.3 Propaganda, World War II and the Cold War

Since the entire *Fallout* series is greatly inspired by World War II and the Cold War, this section will provide a brief overview of how various propaganda techniques were employed during these periods. World War II represents an advanced iteration of propaganda compared to World War I, serving not only as the largest military conflict in history but also as the most extensive propaganda battle. Nazi commander Hermann Göring, during the Nuremberg trials, summarized the psychological foundation of propaganda as an effort based on fear and advanced through patriotism and pride (Soules, 2015, p. 119). This dual appeal to emotion and fear formed the core of Nazi propaganda. Central to this narrative was the creation of “the Others” – both external and internal enemies, with euphemisms, a tool Hitler borrowed from World War I propaganda, used to further boost this strategy. One infamous euphemism, “the final solution,” masked the systematic mass killing of European Jews (Aly, 1999).

Joseph Goebbels, Minister of Propaganda, played a decisive role in Nazi propaganda efforts, leveraging all available technologies to promote Nazism. He controlled the press, radio, film, artistic outputs, and public rallies, dominating public discourse. Trevor-Roper (1978, as cited in Soules, 2015, p. 131) states that he made sure that nothing was heard or seen in any type of media except what he judged useful for immediate political purposes. Goebbels prioritized mobilizing the masses through emotionally charged, simplistic arguments, targeting common people over intellectuals. British propaganda also exploited media, particularly visual imagery. *The Daily Mirror*’s circulation increased from 2.5 million in 1939 to 3 million in 1948 (Historic Newspapers, 2024), partly due to the erotic comic strip Jane, popular among troops. The newspaper also wielded political influence, exemplified by its 1941 “Victory V” campaign, which resonated with Churchill, the public, and European resistance (Soules, 2015, p. 131). While less influential than during World War I, posters still played a significant role in mobilizing civilians on the home front as means of not only conveying information but also reinforcing the will to persevere and sacrifice (Taylor, 2003, pp. 216–217).

Movies were widely used by both sides in World War II as compelling visuals with emotional appeal. Nazis utilized films to promote their ideology and mobilize support, while British and American propaganda employed them to demonize the enemy. Movies were only one aspect of propaganda-driven artistic production, which also included concerts, plays, and exhibitions, proving that warfare extended beyond the battlefield.

On the other hand, the Cold War, a battle of propagandas without direct armed conflict, revolved around ideological supremacy. The USA championed capitalism, while the Soviet Union advocated communism. Propaganda emphasized fear – nuclear war and ideological expansion – while each side asserted the superiority of its system. Fear tactics included technological determinism (nuclear weapons as an irreversible threat), deterrence (matching threats with equivalent weapons), and first-strike capability concerns (Taylor, 2003, p. 253). Both superpowers simplified their conflict into digestible narratives, creating a zero-sum equation of good versus evil, where the other side was portrayed as aggressive, militaristic and repressive, posing a genuine threat to peace and freedom (Taylor, 2003, p. 253). Soviet propaganda, rooted in primal notions of heaven (the USSR) and hell (capitalism), mobilized people effectively, drawing on prior successes. As Rawnsley (1999, p. 5) observes, an efficient information machinery was created in 1917 and it carefully selected and interpreted information in a way demanded by the USSR political masters. All Soviet propaganda was centralized through Agitprop, influencing publishing, media, education, and cultural organizations, crafting an image of the USSR as a blend of “idyll and epic” (Stites, 1999, p. 91).

The USA adopted a sophisticated propaganda strategy during the Cold War, employing educational exchanges, jazz tours, art exhibits, and international visitor programmes to promote, what Snow (1998/2010, p. 64) calls, its core values of freedom, justice, free enterprise, and open dialogue. Both sides targeted domestic, international, and enemy audiences, though propaganda aimed at enemies proved less effective, highlighting the power of “domestic” propaganda (Rawnsley, 1999, p. 35). In the USA, fear of nuclear war, a central aspect of the *Fallout* series, reshaped family roles and daily life, which was then seen as an object of methodical training, testing and correction (Oakes, 1999, p. 74). Propaganda was disseminated through news channels integrated into local media (e.g., *Voice of America*) and Hollywood films, which depicted battles between good and evil, with Russians consistently cast as villains. By contrast, Soviet propaganda largely avoided films, viewing them as unpredictable (Roberts, 1999, p. 120). The “war of propagandas” or “war of ideologies” effectively divided the world into bipolar competitions, proving that propaganda could rival the impact of armed conflict on a global scale – a model that remains relevant for future conflicts and a model which has found its way into video games such as *Fallout*. The USA’s communist enemy in the *Fallout* series is China rather than the Soviet Union, but the principles of propaganda use are essentially the same.

## 4 Aims, corpus and methodology

Having in mind *Fallout’s* overall dystopian overtone (Domsch, 2015), we can assume that the approach to propaganda is largely satirical, but even in such circumstances it does rely on the standard techniques of propaganda which are achieved through different combinations of multimodal elements. Given all this, we set three bigger research questions related to analyzing posters in the *Fallout* game series:

1. What multimodal features are most dominant in propaganda posters in the *Fallout* series?
2. What propaganda techniques are most frequently used (and combined with other techniques) in these posters?
3. What are the relations of multimodal features and propaganda techniques regarding their co-occurrence in these propaganda posters?

In order to do this, we compiled and analyzed a corpus of 70 posters from the six role-playing games from the *Fallout* series: *Fallout: A Post Nuclear Role Playing Game* (Interplay Entertainment, 1997), *Fallout 2: A Post Nuclear Role Playing Game* (Black Isle Studios, 1998), *Fallout 3* (Bethesda Game Studios, 2008), *Fallout: New Vegas* (Bethesda Game Studios, 2010), *Fallout 4* (Bethesda Game Studios, 2015), and *Fallout 76* (Bethesda Game Studios, 2018). The posters were collected from instances of gameplay and from two online archives of *Fallout*-related articles: Nukapedia: The Fallout Wiki (2024) and The Vault – Fallout Wiki (2024), and the collection itself is supposed to contain all posters in the listed games. There were four posters which appeared in both *Fallout 3* and *Fallout 4*, so they were not counted twice in our analyses. We did not include posters which were commercials for products in the game world nor movie posters unless they contained elements of propaganda, so only posters which could potentially contain elements of propaganda were part of this corpus. What made the process easier is the fact that the two online archives we used had already classified the posters into several categories, so we primarily selected those labelled as propaganda posters, but we also checked the remaining ones and included them if they had similar messages as those in the preset list. The collected posters were then subjected to content analysis, where each of them was annotated independently by three raters with regard to the following features:

1. Its primary (the most represented in terms of the poster’s surface area) and secondary color (the second most represented in terms of the poster’s surface area): blue, red, yellow, white, gold, black, gray, orange, green;
2. Text-image relations, where we had four possible labels: text-dominant posters (where the poster could be fully understood based on the text, but not the image alone), image-dominant posters (where the poster could be fully understood based on the image, but not the text alone), complementary posters (where the poster could be understood only by combining the text and the image) and parallel posters (where both the image and the text conveyed the same message and could be interpreted without one another). This approach was largely based on the procedure for testing modality dominance in verbo-pictorial metaphor and metonymy (Tasić and Stamenković, 2015, 2022);
3. The figures of speech in the poster – whether one of the following has been used: metaphor, metonymy, personification, hyperbole (this included visual, verbal or multimodal figures of speech);
4. Font type (serif or sans-serif) and size (regular text or all caps);
5. Iconography: iconographic elements involving people, flags, and the red star;
6. Slogans, i.e., whether a poster included one (such as “Courage Today, Victory Tomorrow!”); and
7. Propaganda techniques: *Appeal to emotion*, *Appeal to fear*, *Band wagon*, *Card stacking*, *Factoids*, *Glittering generalities*, *Name-calling*, *Testimonial*, and *Transfer* (as defined in the theoretical framework above).

The six features belonging to the multimodal aspect of our study were selected for further analysis based on our preliminary survey of the material, which allowed us to see what features could enable a proper differentiation among the posters. When it comes to the seventh feature, we included all the techniques identifiable in our material.

To showcase the way we approached the material, we can take a look at one of the analyzed posters^1^. The primary color in this poster is blue, while the secondary is yellow. The poster is text-dominant, as its main message is conveyed by the text. There were two figures of speech identified in it – one is personification (Uncle Sam stands for the USA), the other is metonymy (the bald eagle and the US flag stand for the USA). The poster uses a sans-serif font, which has both capitalized and non-capitalized letters. The three elements mentioned in the figures of speech sentence constituted the iconography found in this poster. Finally, it contains a slogan and uses the *Band wagon* technique.

The authors of the paper were the three independent raters – one of them is an expert on propaganda, another on multimodality, while the third had knowledge in both areas, and served as the second-pass rater for both disciplines. They first coded the material independently and then joined to finalize the coding decisions. In 15 cases there were disagreements when it came to categorizing certain elements (mostly classifying linguistic elements as slogans and deciding on different figures of speech), and these were resolved in consultations. If lengthy discussions did not ultimately result in complete agreement between all three raters, the majority rule was applied since having an odd number of raters allowed for it. Given the total amount of coded elements (66 posters x 27 variables, all visible in the Supplementary materials), this number is very small. After this, the raters crossed the results within the categories which reflected multimodal features (1–6) and propaganda techniques (7), and then also checked for co-occurrences between these two categories to see whether different propaganda techniques could be related to the use of specific multimodal elements and their various combinations.

## 5 Results

The results explore the persuasive strategies employed in the posters and examine both their multimodal features and the specific propaganda techniques utilized. We begin by exploring the individual multimodal elements present, followed by presenting the identified propaganda techniques and their combined use. Finally, we investigate the link between these two aspects and look at how the multimodal features contribute to and reinforce the effectiveness of the propaganda techniques employed.

### 5.1 Multimodal features in the posters

The analyzed dataset consists of 66 posters, with additional 4 posters which were not counted as they occur in both *Fallout 3* and *Fallout 4*. The biggest number of posters originate from *Fallout: New Vegas* (20 posters), followed by *Fallout 3* (17 posters) and *Fallout 4* (13 + 4 posters). The least representation comes from *Fallout* (1 poster), *Fallout 2* (6 posters), and *Fallout 76* (9 posters). A significant portion of the posters originate from either the real world or the game-world factions and organizations. The *USA* is the most common source (23 posters, 34.8%), followed by *Vault-Tec* (11 posters, 16.7%), *NCR* (8 posters, 12.1%), and *Vault 11* (8 posters, 12.1%). Other sources include *Repcon* (4 posters, 6.1%) and *Free States* (3 posters, 4.5%).

The most common primary color used in posters is *blue* (24 posters, 36.4%), followed by *red* (16 posters, 24.2%) and *yellow* (11 posters, 16.7%). Other colors such as *white* (3 posters, 4.5%), *black* (5 posters, 7.6%), and *gray* (4 posters, 6.1%) appear less frequently, with *orange* and *green* being the least used (1 poster, 1.5%, and 2 posters, 3.0%, respectively). As for secondary colors, *red* is the most frequent (23 posters, 34.8%), followed by *blue* (12 posters, 18.2%) and *yellow* (12 posters, 18.2%). Finally, *black* appears more frequently as a secondary color (9 posters, 13.6%) than as a primary color, usually used for contrast and emphasis. The color co-occurrence analysis reveals strong associations between certain hues. *Blue* and *red* emerge as the most frequent pairing, appearing together on 22 occasions. *Yellow* and *blue* also exhibit a high degree of co-occurrence, a total of 10 times. *Red* and *black* appear together 6 times, and the same goes for *red* and *yellow*.

When it comes to text-image relations, the vast majority of posters (53 posters, 80.3%) are *text-dominant*, which indicates a reliance on slogans and written messages. *Complementary* text-image relations appear in 10 posters (15.2%), while *parallel* relations are much less common, occurring in only 3 posters (4.5%). It is interesting to note that we did not identify any instances of *image-dominant* posters in our dataset.

As for the figures of speech present in the game series (and here expressed multimodally), a total of 29 posters (43.9%) uses multiple figures of speech, while 20 posters (30.3%) use a single figure, and 17 posters (25.8%) use none. *Metaphors* appear in 22 posters (33.3%), while *metonymy* is more common, found in 37 posters (56.1%). *Personification* is used in 15 posters (22.7%), and *hyperbole* in 13 posters (19.7%), while other figures of speech are rarely employed (4 posters, 6.1%). The analysis of figure of speech co-occurrences reveals several key relationships. The most frequent pairing is between *metonymy* and *metaphor*, occurring 10 times. *Hyperbole* also frequently appears with *metonymy* (9 times), as does *personification* with *metonymy* (9 times). *Hyperbole* and *personification* co-occur on 5 occasions.

When it comes to font types, *sans-serif* fonts dominate 51 posters (77.3%), which suggests a preference for clear, bold, easily visible and modern typography. *Serif* fonts appear in 8 posters (12.1%), and *handwriting* styles in 3 posters (4.5%). Additionally, over half of the posters (37 posters, 56.1%) use all capital letters and many of these reinforce their strong propagandistic and persuasive tone. In terms of iconography, 27 posters (40.9%) contain no iconography, while 20 posters (30.3%) use one iconographic element, and 19 posters (28.8%) use multiple. Iconography based on human figures (e.g., Uncle Sam, Lady Liberty) appears in 18 posters (27.3%). The *Red Star* appears in 4 posters (6.1%) and indicates direct references to communism or socialist themes. *Flags* (mostly variants of the US flag) appear in 22 posters (33.3%), while one-third of the posters (22 posters) include slogans.

Table 1 shows a quantitative overview of how *metaphor*, *metonymy*, *personification*, and *hyperbole* in propaganda posters can be related to various multimodal elements coded in our study. *Red* is the dominant primary and secondary color for *metaphor*, *metonymy*, and *personification*, whereas *hyperbole* is more associated with *yellow*. *Text-dominant* relations prevail across all categories, with the highest percentage in *hyperbole* (92.3%). *Sans-serif* fonts are consistently preferred, and capitalized text is most prominent in *hyperbole* (69.2%). The use of *iconography* is most dominant in *metonymy* (81.1%), while the presence of flags is particularly notable in *metonymy* (54.1%) and *personification* (53.3%). Slogans are most frequently employed in *hyperbole* (46.2%) and *personification* (40%).

**Table 1:** Distribution of multimodal features across figures of speech in posters (the number and the percentage show either the most dominant feature or the representation rate if specified).

Figures of Speech →	Metaphor	Metonymy	Personification	Hyperbole
Other Elements ↓				
Poster Source/Author	USA (8, 36.4%)	USA (20, 54.1%)	USA (9, 60%)	USA (3, 23.1%)
Primary color	Red (9, 40.9%)	Red (12, 29.7%)	Blue (6, 40%)	Yellow (6, 46.2%)
Secondary color	Red (7, 31.8%)	Red (12, 29.7%)	Red (5, 33.3%)	Blue (6, 46.2%)
Text-image relations	T.-dom. (16, 72.7%)	T.-dom. (27, 73%)	T.-dom. (12, 80%)	T.-dom. (12, 92.3%)
Font type	Sans-ser. (17, 77.3%)	Sans-ser. (27, 73%)	Sans-ser. (12, 80%)	Sans-ser. (10, 76.9%)
All capital letters	13 (59.1%)	17 (45.9%)	9 (60%)	9 (69.2%)
Iconography used	11 (50%)	30 (81.1%)	10 (66.6%)	11 (84.6%)
Iconography–People	4 (18.2%)	12 (32.4%)	7 (46.7%)	5 (38.5%)
Iconography–Red Star	2 (9.1%)	4 (10.8%)	1 (6.7%)	2 (15.4%)
Iconography–Flags	7 (31.8%)	20 (54.1%)	8 (53.3%)	5 (38.5%)
Slogans used	5 (22.7%)	13 (35.1%)	6 (40%)	6 (46.2%)

We also conducted chi-square tests to examine the relationship between using a figure of speech and employing the elements belonging to different modes, and a set of other relations among different multimodal elements we annotated. We identified the following statistically significant associations between pairs of variables: using *metaphor* and choosing among different *text-image relations* [*χ^2^*(2, *N* = 66) = 6.286, *p* = 0.043], using *metonymy* and using *iconography* in general [*χ^2^*(2, *N* = 66) = 19.214, *p* < 0.001], using *metonymy* and using *iconography involving flags* [χ^2^(1, *N* = 66) = 16.269, *p* < 0.001], using *personification* and choosing among different *text-image relations* [*χ^2^*(2, *N* = 66) = 13.141, *p* = 0.001], using *personification* and using *iconography* in general [*χ^2^*(2, *N* = 66) = 6.180, *p* = 0.046], with those instances involving people being marginally significant [*χ^2^*(1, *N* = 66) = 3.681, *p* = 0.055], but still more significant than the remaining subtypes. Besides this, significant pairs also included: using *hyperbole* and using *iconography* in general [*χ^2^*(2, *N* = 66) = 8.889, *p* = 0.01], *the source* or the author of the poster was significantly associated with the choice of *primary colors* [*χ^2^*(63, *N* = 66) = 104.030, *p* < 0.001], with the general use of *iconography* [*χ^2^*(18, *N* = 66) = 29.844, *p* = 0.039], especially *iconography involving flags* [*χ^2^*(9, *N* = 66) = 26.810, *p* = 0.002].

### 5.2 Propaganda techniques in the posters and combining techniques

The frequency results of different propaganda techniques in the *Fallout* series’ posters reveal a strong reliance on emotionally charged messaging. The most frequently used techniques are *Appeal to emotion* and *Appeal to fear*, both appearing in 28 out of 66 posters (42.4%). The *Band wagon* technique is also relatively common, found in 25 posters (37.9%). On the other hand, techniques such as *Card stacking* (1 poster) and *Testimonial* (1 poster) are very rare. *Factoids* appear in only 3 posters (4.5%), while *Transfer* is present in 9 posters (13.6%), which means that these techniques play a relatively minor role. *Glittering generalities* (17 posters, 25.8%) and *Name-calling* (15 posters, 22.7%) are moderately present. As far as combinations of techniques are concerned, the most common pairings are *Appeal to emotion* and *Band wagon* (10 instances), *Appeal to emotion* and *Glittering generalities* (also 10 instances), *Appeal to fear* and *Band wagon* (9 instances), *Band wagon* and *Glittering generalities* (7 instances), *Appeal to emotion* and *Appeal to fear* (also 7 instances), while *Transfer* co-occurs 7 times with both *Appeal to emotion* and *Glittering generalities*.

### 5.3 Relating multimodal features with propaganda techniques

In Table 2 we present a quantitative overview of rhetorical and visual strategies used in propaganda posters, categorizing them by six key persuasive techniques: *Appeal to emotion*, *Appeal to fear*, *Band wagon*, *Glittering generalities*, *Name-calling*, and *Transfer*. The remaining three techniques were represented in 3 or fewer posters, which is why there was no data available to compare them with other techniques. This is why we omitted them from the table.

**Table 2:** Multimodal choices in propaganda posters in the *Fallout* series: a comparative overview of propaganda techniques (multimodal features per each propaganda technique).

Technique →	Appeal to emotion	Appeal to fear	Band wagon	Glittering generalities	Name-calling	Transfer
Elements ↓						
Poster source/Author	USA (9, 32.1%)	USA (12, 42.3%)	USA (9, 36%)	NCR (4, 23.5%)	USA (8, 53.3%)	USA (5, 55.6%)
Primary color	Blue (13, 46.4%)	Red (10, 35.7%)	Blue (9, 36%)	Blue (6, 35.3%)	Red (9, 60%)	Blue (4, 44.4%)
Secondary color	Red (11, 39.3%)	Black (8, 28.6%)	Red (10, 40%)	Red/Blue (5, 29.4%)	Black (5, 33.3%)	Red (5, 55.6%)
Text-image relations	T.-dom. (22, 78.6%)	T.-dom. (20, 71.4%)	T.-dom. (22, 88%)	T.-dom. (13, 76.5%)	T.-dom. (10, 66.7%)	T.-dom. (8, 88.9%)
Figures of speech used	24 (85.8%)	24 (85.8%)	21 (84%)	13 (76.5%)	11 (73.3%)	9 (100%)
Metaphor used	8 (28.6%)	13 (46.4%)	6 (24%)	4 (23.5%)	9 (60%)	2 (22.2%)
Metonymy used	18 (64.3%)	17 (60.7%)	15 (60%)	11 (64.7%)	11 (73.3%)	8 (88.9%)
Personification used	6 (21.4%)	9 (32.1%)	8 (32%)	4 (23.5%)	3 (20%)	5 (55.6%)
Hyperbole used	5 (17.9%)	6 (21.4%)	5 (20%)	6 (35.3%)	1 (6.7%)	5 (55.6%)
Font type	Sans-ser. (22, 78.6%)	Sans-ser. (21, 75%)	Sans-ser. (20, 80%)	Sans-ser. (12, 70.6%)	Sans-ser. (9, 60%)	Sans-ser. (7, 77.8%)
All capital letters	10 (35.7%)	16 (57.1%)	14 (56%)	6 (35.3%)	13 (86.7%)	3 (33.3%)
Iconography used	21 (75%)	14 (50%)	21 (84%)	11 (64.7%)	8 (53.3%)	6 (66.7%)
Iconography–People	13 (46.4%)	5 (17.9%)	11 (44%)	5 (29.4%)	0 (0%)	5 (55.6%)
Iconography–Red Star	1 (3.6%)	3 (10.7%)	3 (12%)	1 (5.9%)	2 (13.3%)	0 (0%)
Iconography–Flags	14 (50%)	5 (17.9%)	11 (44%)	8 (47.1%)	5 (33.3%)	7 (77.8%)
Slogans used	11 (39.3%)	6 (21.4%)	11 (44%)	8 (47.1%)	2 (13.3%)	5 (55.6%)

Finally, we also conducted chi-square tests to examine the relationship between using a particular propaganda technique and employing the elements belonging to different modes, i.e., to assess whether the choice of technique favored some of the options we coded (particular colors, figures of speech, iconography, etc.). In several instances we found statistically significant associations between pairs of variables, and these were: *Appeal to emotion* and the general use of *iconography* [*χ*^2^(2, *N* = 66) = 14.65, *p* = 0.001], *Appeal to emotion* and the use of *iconography involving people* [*χ*^2^(1, *N* = 66) = 8.997, *p* = 0.003], *Appeal to emotion* and the use of *iconography involving flags* [*χ*^2^(1, *N* = 66) = 6.079, *p* = 0.014]; *Appeal to fear* and the choice of *primary color* [*χ^2^*(7, *N* = 66) = 18.528, *p* = 0.010], *Appeal to fear* and the use of *iconography involving flags* [*χ^2^*(1, *N* = 66) = 5.242, *p* = 0.022], *Band wagon* and the general use of *iconography* [*χ^2^*(2, *N* = 66) = 11.695, *p* = 0.003], *Band wagon* and the use of *iconography involving people* [*χ^2^*(1, *N* = 66) = 5.677, *p* = 0.017], *Glittering generalities* and the use of *all capital letters* [*χ^2^*(1, *N* = 66) = 4.009, *p* = 0.045], *Name calling* and the choice of *primary color* [*χ^2^*(7, *N* = 66) = 16.468, *p* = 0.021], *Name calling* and the use of *metaphor* [*χ^2^*(1, *N* = 66) = 6.212, *p* = 0.013], *Name calling* and the use of *all capital letters* [*χ^2^*(1, *N* = 66) = 7.382, *p* = 0.007], *Name calling* and the general use of *iconography* [*χ^2^*(2, *N* = 66) = 9.143, *p* = 0.010], *Name calling* and the use of *iconography involving people* [*χ^2^*(1, *N* = 66) = 7.279, *p* = 0.007], *Transfer* and the use of *metonymy* [*χ^2^*(1, *N* = 66) = 4.559, *p* = 0.033], *Transfer* and the use of *personification* [*χ^2^*(1, *N* = 66) = 6.395, *p* = 0.011], *Transfer* and the use of *hyperbole* [*χ^2^*(1, *N* = 66) = 8.472, *p* = 0.004], *Transfer* and the general use of *iconography* [*χ^2^*(2, *N* = 66) = 9.489, *p* = 0.009], *Transfer* and the use of *iconography involving people* [*χ^2^*(1, *N* = 66) = 4.203, *p* = 0.040], and *Transfer* and the use of *iconography involving flags* [*χ^2^*(1, *N* = 66) = 9.263, *p* = 0.002].

## 6 General discussion

In this section we will try to directly address the three research questions set at the beginning of our study based on the obtained results presented above. Besides this, we will try to briefly outline how our analysis contributes to *Fallout* scholarship, propaganda studies and multimodality.

### 6.1 What multimodal features are most dominant in propaganda posters in the *Fallout* series?

In our analysis we annotated six different multimodal features of the examined posters. These were: colors, text-image relations, figures of speech, font types, iconography, and slogans. Starting with the first of the above features, the most dominant colors in the analyzed posters were blue and red, both when present separately or in combination with each other. As the colors most easily associated with the USA, primarily stemming from the American flag, it is more or less self-evident that they are used to convey patriotic or military themes, which are in line with the games’ primary subject matter. However, red is also used for a different effect, particularly in combination with black. On its own, it is the color of the franchise’s main antagonistic ideology, i.e., communism, and it is sometimes used, often together with black, to invoke fear, distrust, or animosity. Next, even though our corpus may at first appear to be predominantly visual, the most prominent text-image relation by far was the one in which the poster could be fully understood based on the text only. In fact, there were no examples of image-dominant posters in the entire analyzed dataset. As already mentioned, we believe that this comes from the principal function of these posters, where they serve as sources of information and propaganda, which is much more easily and effectively communicated via text rather than images that could perhaps be more prone to different interpretations, leading to the loss of the intended message.

The analysis of the third feature, the multimodal figures of speech, showed that almost three quarters of the collected posters possess some form of figurative language, with *metonymy* being the most common figure employed. It is also found in the most common of pairings, together with *metaphor*, further emphasizing the role of *metonymy* as a specific conceptual shortcut, regularly used in the representation of political or ideological entities that the games abound in (see Tasić, 2023, for the analysis of verbal, pictorial, and multimodal metonymy in political discourse). The same percentage of posters is related to the next feature as well, in that all capital letters are present in 37 *Fallout* propaganda posters analyzed in our study. Along with an even more dominant statistic that shows that *sans-serif* fonts are used in 51 posters, these two findings imply that said font types and sizes are utilized to make messages clearer, stronger, and more convincing. This is also fully in line with the posters’ primary propagandistic function.

This function is further conspicuously underlined by the final two multimodal features. As far as iconography is concerned, when present, it is mostly in the form of flags, particularly the US flag and its variants indigenous to the game-world, or human figures, again mainly personifying the USA. On the other hand, slogans that appear in exactly one third of the analyzed posters often accompany such figures, almost exclusively with the aim of further stressing the propagandistic nature of the entire multimodal composition. The use of all these multimodal features in the games’ world-building elements evidently shows how different real-life propaganda strategies and methods are adapted to best serve the franchise’s narrative.

### 6.2 What propaganda techniques are most frequently used (and combined with other techniques) in these posters?

As expected, the most frequently used techniques are *Appeal to emotion* and *Appeal to fear*. These results are directly connected to the primary purpose of propaganda – to affect emotions and not reason. These two techniques are the most effective for provoking desired emotional reactions and manipulating fear. In the context of the *Fallout* universe we can say that these tactics were employed to evoke strong emotional responses and, in most cases, to mock such messages. This is in line with the game’s overarching themes of survival, loyalty, and ideological manipulation. The *Band wagon* technique is also relatively common, and it can indicate an emphasis on collective action and conformity, which corresponds to the game’s depictions of factions, societal structures, and recruitment efforts. Also, homogeneity of society or certain groups is a central theme of every war propaganda. *Glittering generalities* and *Name-calling* are moderately present, which points to the use of virtue-laden slogans and derogatory language to shape ideological perspectives. The propaganda messages constructed either to degrade the other side or celebrate our own actions are also essential to war propaganda.

In addition, these emotionally charged fear-based propaganda techniques are very often combined to increase their persuasive potentials. The most common pairing, *Appeal to emotion* and *Band wagon*, likely signals the importance of emotional engagement in maintaining a sense of collective belonging. Such posters encourage the viewer to align with a group or movement, often leveraging patriotic or survivalist sentiments to persuade individuals to conform. This combination suggests that, given the over-emphasis of these techniques as a result of satire, *Fallout’s* propaganda leans heavily on social pressure and emotional manipulation, which is in line with the in-universe totalitarian and war-driven societies. Similarly, *Appeal to emotion* and *Glittering generalities* work together to construct idealized narratives that are emotionally resonant but often vague in their actual promises. The frequent co-occurrence of *Appeal to fear* and *Band wagon* can stress the dual strategy of fear-based persuasion and group conformity. Such posters usually depict external threats while simultaneously urging the audience to rally together. This mirrors Cold War-era propaganda tactics that emphasized national unity against a common enemy and reinforces *Fallout’s* satirical critique of the mid-20th-century propaganda. *Band wagon* also co-occurs with *Glittering generalities* and this combination likely means a lack of substance in the argument being presented and a reliance on emotional appeal rather than factual evidence. Another notable trend is the combination of *Appeal to emotion* and *Appeal to fear*, which means that some posters employ both positive and negative emotional triggers. The frequent presence of *Transfer* in co-occurrences, particularly with *Appeal to emotion* and *Glittering generalities*, indicates that some posters associate strong emotional or ideological values with authoritative symbols. This suggests that official government, military, or Vault-Tec imagery is often employed to lend credibility to the propaganda messages, much like real-world wartime and corporate propaganda. All in all, the overall distribution of these techniques shows the game’s satirical approach to propaganda – it still employs traditional methods while maintaining a degree of sarcasm and critique.

### 6.3 What are the relations of multimodal features and propaganda techniques regarding their co-occurrence in these propaganda posters?

The relationship between propaganda techniques and multimodal elements in *Fallout* posters reveals distinct patterns in how persuasion is aided or achieved through visual and textual means. Across all techniques, text-dominant compositions seem to be the norm, with *Band wagon* and *Transfer* relying on explicit verbal messaging the most, while *Sans-serif* fonts dominate. Color choices reflect the emotional tone of each technique: *Appeal to emotion* and *Band wagon* favor blue, which may evoke trust and unity as in most cases these are derived from the colors of the US flag as we have already seen, whereas *Appeal to fear* and *Name-calling* lean heavily on red and black, which can reflect urgency, danger and hostility. Figures of speech differentiate these techniques – while metonymy is prevalent across all categories as stated above, peaking in *Transfer*, metaphor use varies, appearing most in *Name-calling*, where comparisons to negative imagery can lead to adversarial messaging, and least in *Transfer*, which instead prioritizes direct symbolic associations. *Personification* is particularly notable in *Transfer*, where in some cases abstract entities are given human-like traits to boost ideological appeal, while *Hyperbole* reaches its peak in *Glittering generalities* and *Transfer*. On the other hand, iconography serves different strategic purposes – flags are overwhelmingly used in *Transfer* and *Appeal to emotion* to reinforce nationalistic, faction-related or ideological belonging, while human imagery is most common in *Band wagon* and *Transfer*, which can be related to creating a sense of collective participation or authority. Notably, the red star, a direct ideological marker, is most frequently found in *Appeal to fear* and *Band wagon*. The role of typography and slogans can also be related to some techniques. *Name-calling* stands out as likely the most aggressive, as it relies quite a lot on capitalized text. At the same time, with this technique we find high metaphor density and minimal iconography, which might indicate a stark and confrontational tone. *Transfer* relies on hyperbolic language, heavy metonymy, and the use of national symbols to establish ideological connections. Finally, slogans are most prevalent in *Band wagon* and *Transfer*, which points to the role of repetition and catchphrases in mobilizing audiences.

Given all this, we can say that this study contributes to *Fallout* scholarship by examining how the game’s in-universe propaganda posters adapt real-world ideological strategies to support narrative and world-building. It adds to propaganda studies by exploring how traditional techniques function in a fictional digital context, often combined to evoke emotional responses and reinforce ideological messages. The satirical framing of these techniques reflects familiar wartime rhetoric while also commenting on it. From the perspective of multimodal scholarship, the study focuses on several features, including color, text-image relations, figures of speech, font types, iconography, and slogans, and analyzes how these interact with specific propaganda techniques. By linking these multimodal elements with persuasive strategies, the study offers insight into how propaganda functions across modes, not only in real-world settings but also in fictional media.

## 7 Conclusion

The *Fallout* series uses propaganda techniques and multimodal elements to construct its dystopian, retro-futuristic world. The biggest difference, of course, is the fact that the main reason for using propaganda in the first place is satirical. The use of multimodal elements in *Fallout* posters is similar to historical propaganda strategies, especially those used during wartime and the Cold War. The presented analysis was an attempt to combine the investigation of multimodal features of the compiled corpus of posters from six *Fallout* games with the examination of a set of specific real-world propaganda techniques implemented within them. The most important findings show how the specific use of these features appropriates and adapts real-world propaganda strategies, providing further support to the intricate in-game world-building process.

However, we must also list several limitations which should be acknowledged regarding our current approach. One of the primary constraints is the relatively small dataset of 66 posters. While sufficient for identifying general trends, this sample size limits the statistical significance of some findings. A larger corpus could provide a more comprehensive representation of the different propaganda techniques and multimodal strategies present in the video games selected using a set of criteria. This corpus, however, contained all posters in the series which were relatable to propaganda, so there was no way to expand it. The study therefore primarily focuses on qualitative analysis with quantitative elements, which, while effective for pattern recognition, does not allow for broader generalizations beyond this specific game franchise. Another limitation lies in the subjective nature of categorizing certain multimodal features, such as figures of speech and propaganda techniques, which we tried to solve by reaching a consensus in all cases where we did not agree on the category. Lastly, this study does not account for player reception and interpretation, which could provide further insights into how these posters function within gameplay and narrative and the way they are experienced.

As we noted, future studies could expand on this research by analyzing a larger dataset of posters, which could be expanded with posters from other games. This would allow for comparative analyses with different video games and with real-world historical propaganda posters, particularly from different cultural contexts. In line with the limitations, research focusing on player reception could explore how audiences interpret and engage with these posters, i.e., check whether they recognize the satire, whether they are influenced by the propaganda themes, or whether they find these visuals as something that contributes to ideological narratives. Moreover, for each poster used in this study we could provide an in-depth qualitative analysis, with extracts of gameplay, and include considerations of the semiotic aspects and the rhetorical strategies within the qualitative variables. Given the nature of the posters analyzed, they could also be subject to an approach based on social semiotics (e.g., Pérez-Latorre et al., 2016) or digital propaganda studies (e.g., Lin, 2024; Wooley, 2022). Another possible area of expansion would be a study of interactive propaganda elements within video games beyond static posters, such as radio broadcasts, dialogue, and mission-based persuasion strategies. Finally, exploring the evolution of propaganda techniques in video games over time (from early gaming history to contemporary trends) could offer a longitudinal perspective on how digital propaganda adapts to new media forms and perhaps to some changes in our societies.

## Data Availability

The original contributions presented in the study are included in the article/Supplementary material, further inquiries can be directed to the corresponding author/s.

## Footnotes

^1^ https://fallout.fandom.com/wiki/Fallout_and_Fallout_2_posters?file=Fo2_Defend_Your_Country_Orig_Poster.jpg
